# Cellular Blue Nevus Diagnosed following Excision of Melanoma: A Challenge in Diagnosis

**DOI:** 10.1155/2016/8107671

**Published:** 2016-05-26

**Authors:** Nives Jonjić, Andrea Dekanić, Nedeljka Glavan, Larisa Prpić-Massari, Blaženka Grahovac

**Affiliations:** ^1^Department of Pathology, Rijeka University School of Medicine, Braće Branchetta 20, 51000 Rijeka, Croatia; ^2^Department of Pediatric Surgery, Rijeka University Hospital Center, Rijeka, Croatia; ^3^Department of Dermatovenerology, Rijeka University Hospital Center, Rijeka, Croatia

## Abstract

A case of a 41-year-old woman with a history of nodular melanoma (NM), associated with an indurated dome-shaped blue-black nodule with a diameter of 1.2 cm in the gluteal region, is presented. Clinical diagnosis of the lesion, present from birth, was blue nevus. Recently, the nodule has been showing a mild enlargement and thus complete resection was performed. Histological analysis revealed a pigmented lesion with an expansive pattern of extension into the dermis and the subcutaneous adipose tissue. The lesion displayed an alveolar pattern as well as a pigmented dendritic cell pattern. The histology was consistent with cellular blue nevus (CBN); however, the history of NM which was excised one year earlier, as well as the clinical information about the slow growing lesion, included a differential diagnosis of CBN, borderline melanocytic tumor, and malignant blue nevus. Additional immunohistochemical (HMB-45, p16, and Ki-67) and molecular (BRAF V600E mutation) analyses were performed on both lesions: the CBN-like and the previously excised NM. Along with lesion history and histological analyses, p16 staining and BRAF were useful diagnostic tools for confirming the benign nature of CBN in this case.

## 1. Introduction

Blue nevi are a subset of melanocytic proliferations of embryonic neural crest origin containing cells which are similar to dendritic melanocyte precursors [1]. The nature and developmental biology of blue nevus and its variants are so far not very clearly understood. Some of its rare variants do present diagnostic difficulties because it is hard to differentiate between benign and malignant blue nevi and to differentiate them from other melanocytic lesions [2]. Cellular blue nevus (CBN) differs from classic blue nevus since it exhibits a cellular appearance and presents itself with subcutaneous infiltration, intensive pigmentation, and a large size. Thus it can be wrongly diagnosed as melanoma due to atypia criteria that may be present [3–6].

In order to address the above problem immunohistochemistry can be a useful tool in the diagnosis of some cases of melanoma, and markers such as S-100, HMB-45, Melan A, MITF, Ki-67, and p16 have been found to be useful in distinguishing between benign and malignant melanocytic lesions [7, 8].

Some studies have reported that the activation of the mitogen-activated protein kinase (MAPK) signaling pathway, as a result of the somatic mutation of BRAF, is a crucial event in the development of melanoma [9, 10]. However, which specific mutation is a precursor of the disease is still controversial since a number of studies concluded that the mutation of BRAF or NRAS genes are not specific for the progression of nevus to melanoma [11–13]. Other studies showed that the mutational activation of the RAS/RAF/MAPK pathway in nevi is a critical step in the initiation of melanocytic neoplasia; however this alone seems to be insufficient for melanoma tumorigenesis [14].

In the present report a case of CBN, the less common blue nevi lesions that can often be confused with melanoma especially when diagnosed following the excision of melanoma and dysplastic nevi as in the case of our patient, is described.

## 2. Case Report

A 41-year-old woman presented to surgery with a pigmented plaque on the gluteal region which was present from birth. One year ago she had pigmented lesion operated on from the anterior part of the chest. The histological examination revealed melanoma of nodular type. The Breslow thickness was 3 mm and Clark level II/III. Ulceration was present. Mitotic rate was 5 mitoses/mm^2^. The patient underwent wide reexcision of the primary tumor site and sentinel lymph nodes biopsy. Five sentinel lymph nodes were immunohistochemically analyzed and no metastasis was detected. Final pathologic staging was pT3b, N0. Patient did not receive any therapy but was regularly controlled by oncologist and dermatologist. In the mean time she had five pigmented lesions operated on from different site of trunk and leg. The excised biopsy specimens revealed diagnosis of nevi and dysplastic nevi.

**Figure 1:**
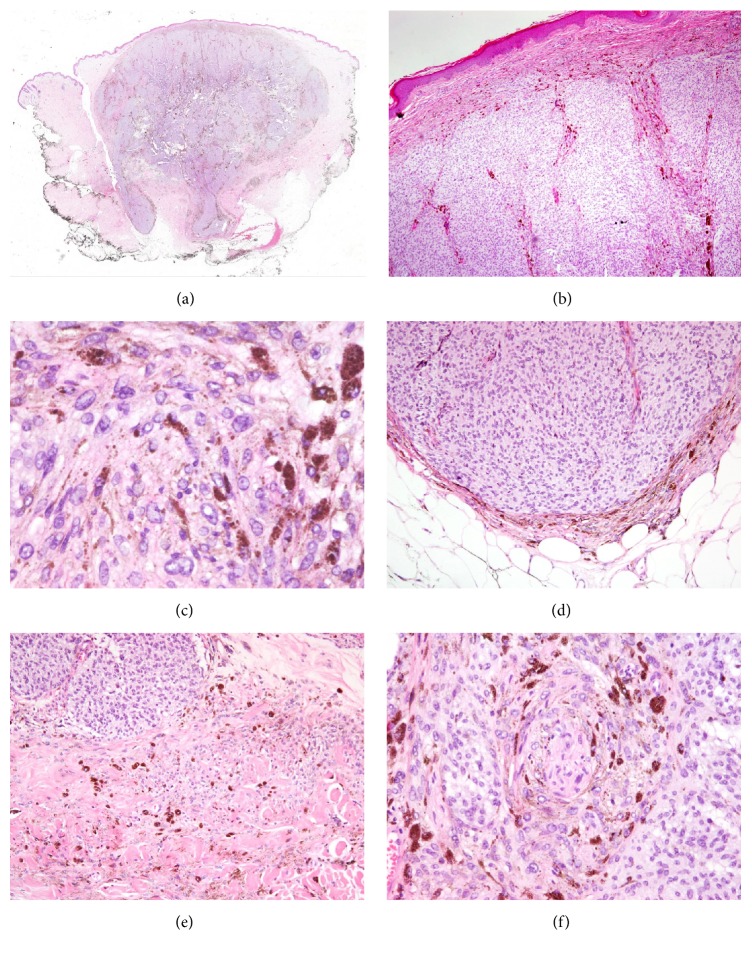
A pigmented lesion consisted with cellular blue nevus with an expansive pattern of extension into dermis and subcutaneous adipose tissue (a) and a spared papillary dermis (b). Cellular islands of closely aggregated spindle shaped cells with ovoid nuclei revealed a mild nuclear enlargement, a mild pleomorphism, and prominent small nucleoli (c). The deepest boundary of the tumor was a pushing border (d) with foci of more irregular infiltrative borders (e). Perineural invasion was observed (f).

**Figure 2:**
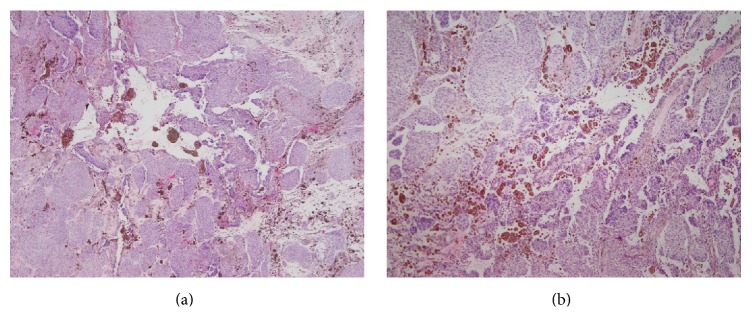
A central part of pigmented lesion with cystic degeneration, small foci of hemorrhage (a), and multinucleated cells that are a common finding in cellular blue nevus (b).

Dermoscopic examination of gluteal pigmented plaque revealed a homogenous, blue-white structure in the region. In the absence of any other dermoscopic structures a clinical diagnosis of blue nevus was established. Recently, the lesion presented with a slow growth. Considering the history of melanoma operation of the patient, the lesion was excised and a biopsy specimen was fixed in 10% buffered-formalin and embedded in paraffin. Paraffin sections of 5 *μ*m were stained with haematoxylin-eosin. Histological analysis revealed a pigmented lesion with an expansive pattern of extension into dermis and subcutaneous adipose tissue (Figure 1(a)). The deepest boundary of the tumor was a pushing border with mild fibrosis. The lesion was composed of an alveolar pattern and a pigmented dendritic cell pattern. No junctional or dermal banal nevus component was present and the papillary dermis was spared (Figure 1(b)). Cellular islands of closely aggregated large spindle shaped cells with ovoid nuclei were surrounded with abundant melanin. A closer inspection revealed a mild nuclear enlargement, an increased nuclear-to-cytoplasmic ration, a mild pleomorphism, and prominent nucleoli without any evident mitotic figure (Figure 1(c)). A magnification assessment has shown that the tumor had a blunt “pushing-type” border with a sharp demarcation line between the tumor and the dermis or subcutaneous tissue (Figure 1(d)). However, in some foci the tumor showed more irregular infiltrative borders (Figure 1(e)). Perineural invasion was present (Figure 1(f)). Tumor necrosis was not observed but in the central part of the lesion a cystic degeneration was present (Figure 2). The morphology was consistent with CBN but somehow suspicious, especially taking into consideration the history of nodular melanoma operation a year before. Therefore, additional immunohistochemical (HMB-45, p16, and Ki-67) and molecular (BRAF V600E mutation) analyses were performed on CBN-like and NM.

**Figure 3:**
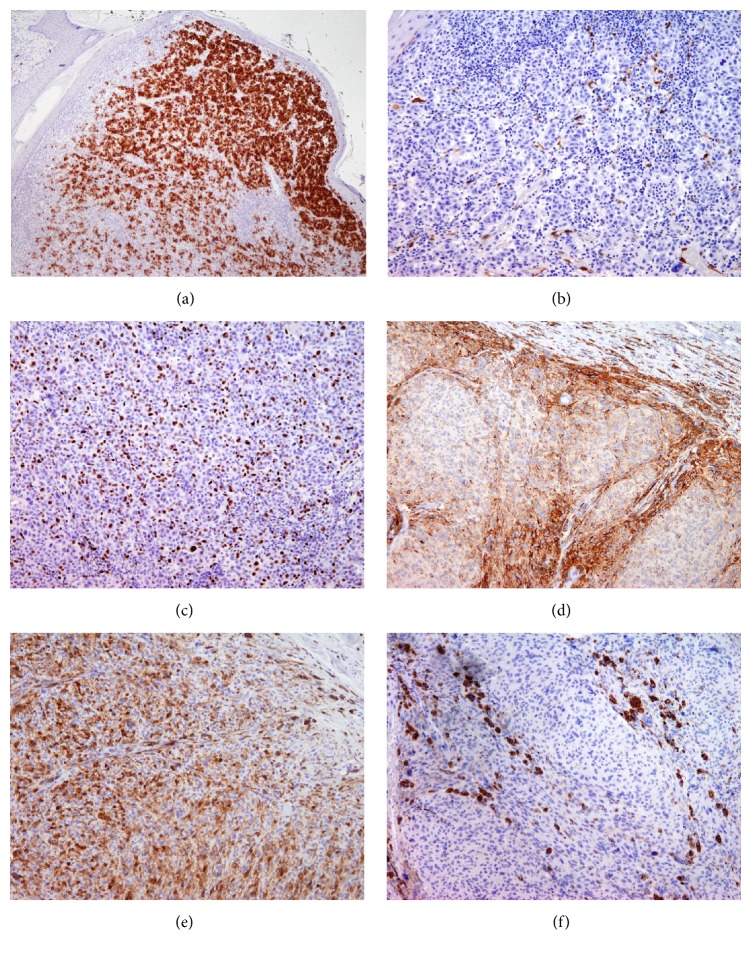
Immunohistochemical staining of nodular melanoma (NM) and cellular blue nevus (CBN) with HMB-45, p16, and Ki-67: HMB-45 was strongly positive in NM (a) while in CBN the reactivity was mostly present at the periphery of alveolar nets (d); p16 was negative in NM (b) but present in the majority of tumor cells in CBN (e); Ki-67 was positive in 33% of melanoma cells (c) and negative in CBN (f).

Immunostaining with HMB-45 was strongly positive in more than 80% of tumor cells in NM (Figure 3(a)) while reactivity was focally present at the periphery of alveolar nets in CBN-like (Figure 3(d)). p16 was present in more than 60% of tumor cells (Figure 3(e)) in CBN-like while melanoma cells were negative (Figure 3(b)). Ki-67 staining was almost negative in CBN (<0.5%) (Figure 3(f)) while the mitotic index in NM amounted to 33% (Figure 3(c)). Molecular analysis confirmed a mutation in BRAF V600E in melanoma cells but not in CBN-like. Based on these findings a histological diagnosis of CBN was confirmed.

## 3. Discussion

The current case presents CBN as a less common form of blue nevus that additionally contained some atypical features which required a differentiation from malignant blue nevus, especially because the patient presented with a history of excised melanoma. Most melanomas are thought to arise de novo; however, they may develop in association with a preexisting benign melanocytic lesion. In addition, the term “malignant blue nevus” has been applied most often to melanomas that arise in the background of cellular blue nevus [15–17].

In the literature there are some reported cases of melanoma that arose in a congenital CBN in older patients (73 and 69 years) with a predilection for the scalp area [18, 19]. Published data indicates that melanoma arising in association with a preexistent CBN typically shows a stable size or a very slow growth before becoming clinically relevant. The patients usually seek medical care due to an increase in size as it was the case with our patient.

In general, CBN is not common and CBN-related melanoma cases are exceedingly rare, making it difficult for pathologist and clinicians to elucidate the biological nature and the malignancy potential of these cases. According to some authors CBN-related melanoma is a low-grade malignancy [20] with a metastatic pattern and behavior comparable to other types of melanoma [21]. In contrast, other studies describe the malignant form of CBN as a highly aggressive, malignant, and often lethal tumor, with a propensity for metastasis to the lymph nodes and lungs [19, 22].

There is no consensus regarding the classification or definite diagnostic criteria for CBN, atypical CBN, and malignant blue nevus [6]. The histology of malignant blue nevus includes a sheet-like growth pattern with a loss of normal biphasic or alveolar architecture, as seen in CBN, and variable necrosis. These tumors are more diagnostically challenging, because atypical cytologic features are not directly juxtaposed to bland appearing nevus cells. However, under closer inspection at least several features that fulfill some of the architectural and cytomorphological concepts of malignancy are observed, such as infiltrative borders, necrosis, frequent mitosis, nuclear pleomorphism and hyperchromasia, and epithelioid cell morphology. Some authors have suggested that the most important criteria for distinguishing benign from malignant CBN are the presence of widespread necrosis [23] while others think that necrosis is not a very sensitive feature for the diagnosis of malignant blue nevus. Furthermore, those authors believe that necrosis should be distinguished from the areas of liquefactive degeneration that is commonly associated with cystic degeneration, myxoid change, and edema in CBN [19]. In our case a cystic degeneration present in the lesion was the reason for the mild increase, as noticed by our patient.

Immunohistochemical stains can be of great value in the assessment of challenging melanocytic neoplasms. In our case HMB-45 primarily labeled melanoma cells while melanocytes in CBN presented a loss of HMB-45 expression with a progressive descent into the dermis. This finding is in contrast with a previously reported case study in which all cases of CBN were strongly HMB-45 positive [24]. The proliferation marker, Ki-67, in our case was almost zero in comparison to melanoma cells. This finding supported the diagnosis of benign CBN, although some studies indicate a low mitotic rate for malignant blue nevus [22] and a significant mitotic activity in benign CBN [19]. A loss of p16, since this is one of the proteins that regulates the Gi/S checkpoint of the cell cycle, has been documented to occur in melanoma [25]. Thus p16 may be a potentially helpful marker in differentiating atypical melanocytes nevi from melanoma [8]. In our case p16 has been absent in melanoma compared to CBN. This finding is supported by other studies which concluded that p16 staining may be useful in distinguishing between a benign and malignant nature of CBN [26].

Recent data show that mutations in genes responsible for common nevi or melanomas such as BRAF, NRAS, or c-kit are actually rare in blue nevi [11]. In addition, BRAF V600E and GNA11 exon 5 mutations were found only in malignant blue nevus but were not present in atypical CBN or CBN [27]. In our case this was confirmed and mutations in BRAF V600E in CBN were not present. According to the literature, benign and malignant blue nevi harbor frequent mutations in the G*αρ* class of the G-protein *α* subunits, namely, the Gna*ρ* and GNA11 proteins [28]. It is generally accepted that genetic or epigenetic changes which play a role in the transformation of nevi to melanomas are still not identified. Discordant BRAF gene status between melanocytic lesions in our patient, in combination with other criteria, was useful for making the diagnosis of benign CBN.

In conclusion, the diagnostic evaluation of a blue lesion should always rely on the integration of all data, especially clinical findings and dermatoscopic features. Lesions showing an increase in size, especially with a history of melanoma, should always be excised and histologically examined to give a correct diagnosis and avoid the risk of misclassification.
